# Neutrophils and Lymphocytes: Yin and Yang of Lung Fibrosis and Patient Outcome in Diffuse Interstitial Lung Diseases

**DOI:** 10.3390/biomedicines12112439

**Published:** 2024-10-24

**Authors:** Erika M. Novoa-Bolivar, José A. Ros, Sonia Pérez-Fernández, José A. Campillo, Ruth López-Hernández, Rosana González-López, Almudena Otalora-Alcaraz, Cristina Ortuño-Hernández, Lourdes Gimeno, Inmaculada Ruiz-Lorente, Diana Ceballos-Francisco, Manuel Muro, Pablo Martínez-Camblor, Alfredo Minguela

**Affiliations:** 1 Immunology Service, Virgen de la Arrixaca University Clinical Hospital (HCUVA), Biomedical Research Institute of Murcia (IMIB), 30120 Murcia, Spain; e.m.novoab@hotmail.com (E.M.N.-B.); josea.campillo@carm.es (J.A.C.); ruth.lopez2@carm.es (R.L.-H.); rosana13a@hotmail.com (R.G.-L.); almudena.o.a@gmail.com (A.O.-A.); cristina.ortunoh@um.es (C.O.-H.); lourdes.gimeno@carm.es (L.G.); irl_98@hotmail.com (I.R.-L.); dceballosf@gmail.com (D.C.-F.); manuel.muro@carm.es (M.M.); 2 Pneuomology Service, Virgen de la Arrixaca University Clinical Hospital (HCUVA), Biomedical Research Institute of Murcia (IMIB), 30120 Murcia, Spain; jarl77@yahoo.es; 3 Department of Statistics and Operations Research and Mathematics Didactics, University of Oviedo, 33007 Asturias, Spain; perezsonia@uniovi.es; 4 Human Anatomy Department, University of Murcia, 30100 Murcia, Spain; 5 Department of Biomedical Data Science, Geisel School of Medicine at Dartmouth, 7 Lebanon Street, Suite 309, Hinman Box 7261, Hanover, NH 03755, USA; pablo.martinez-camblor@hitchcock.org

**Keywords:** lung fibrosis, BAL lymphocyte and neutrophils, interstitial lung disease, idiopathic pulmonary fibrosis, patient outcome, flow cytometry

## Abstract

**Objective**: Antifibrotics can improve the outcome of patients with idiopathic pulmonary fibrosis (IPF) and other fibrosing interstitial lung diseases (F-ILDs), but predictive biomarkers at diagnosis are needed to guide the use of immunomodulating and antifibrotic therapies. **Methods**: Flow cytometry quantification of lymphocytes and neutrophils in bronchoalveolar lavage (BAL) of 145 IPFs, 561 non-IPF-ILDs (125 F-ILDs), and 112 BAL controls were retrospectively correlated with the incidence of fibrosis and third-quartile overall survival (Q3–OS). **Results**: The incidence of IPF was directly proportional (9.6%, 22.2%, and 42.6%, *p* < 0.001) to BAL neutrophil counts (<5%, 5–15%, and >15%), but inversely proportional (34.1%, 18.6%, and 8.8%, *p* < 0.001) to BAL lymphocyte counts (<7%, 7–20%, and >20%). Elevated neutrophils (>5%) with low lymphocytes (<7%) were associated with an increasingly higher incidence of IPF (10.0–56.3%, *p* < 0.001) in patients aged 40 to 80, compared to the rest of patients (13.0–17.1%). Lymphocytes >20% compared to lymphocytes <7% strongly protected patients with neutrophils >15% (59.7% vs. 20.7%, *p* < 0.001) from IPF. In contrast, the incidence of F-ILD was not clearly related to BAL lymphocyte/neutrophil counts. Although, IPF and F-ILD showed a shorter Q3–OS (1.8 ± 0.3 and 4.6 ± 0.8 years; *p* < 0.001) than non-fibrotic-ILDs (11.1 ± 1.3 years), lymphocyte and neutrophil counts were associated with a longer and shorter Q3–OS of non-fibrotic-ILDs (*p* < 0.03) and F-ILDs (*p* < 0.04), respectively, but not with a Q3–OS of IPF patients (*p* < 0.708). Corticosteroids in patients with fibrosis showed a shorter Q3–OS than other immunomodulators (2.4 ± 0.3 vs. 4.0 ± 1.8 years, *p* = 0.011). **Conclusions**: Accurate counting of BAL lymphocytes and neutrophils by flow cytometry in ILD patients at diagnosis could help guide immunomodulatory and antifibrotic therapies.

## 1. Introduction

The term interstitial lung disease (ILD) describes a large and heterogeneous group of disorders affecting the lung parenchyma with overlapping clinical, radiographic, and histopathologic manifestations that commonly involve the pulmonary interstitium and, less frequently, also the alveolar and vascular epithelium. Repair processes are involved in the disease, with varying degrees of inflammation and fibrosis [1,2,3], which can be progressive in some cases [4,5,6]. Features of progressive fibrosing ILD (PF-ILD) include a decline in lung function as measured by forced vital capacity (FVC) or diffusing capacity of the lung for carbon monoxide (DLCO), radiographic progression, or worsening symptoms despite treatment [7]. Idiopathic pulmonary fibrosis (IPF), the most typical PF-ILD, is characterized by a pattern of usual interstitial pneumonia (UIP) on high-resolution computed tomography (HRCT) or histopathology and it is not associated with an identifiable etiology [8]. Risk factors for IPF include increasing age, oxygen use at rest, lower or a decline in FVC, and lower DLCO [9], but the clinical course can be complicated by episodes of acute respiratory deterioration or acute exacerbations [10]. Many studies have described that a diverse proportion of fibrosis and progressive fibrosis can also take place among connective tissue disease-related (CTD)-ILD, unclassifiable-ILD, idiopathic interstitial pneumonia, and chronic hypersensitivity pneumonitis (HP), or sarcoidosis [11,12,13,14,15]. Risk factors for non-IPF PF-ILDs include UIP pattern, body mass index, oxygen desaturation during the 6 MWT, and lung function parameters such as FVC and DLCO [14].

Different cells of the immune system are involved in the pathophysiology of ILD. Macrophages play a significant role in fibrosing lung diseases, although their roles may vary in distinct inflammatory microenvironments or with the composition of macrophage subpopulation types [16,17,18]. Neutrophils are also capable of triggering inflammatory mechanisms and lung fibrogenesis [19]. Neutrophil elastase [20], neutrophil extracellular traps [21], and the balance of matrix metalloproteinases and tissue inhibitors of metalloproteinases [22] contribute to fibrosis and to fibrosis progression. Neutrophil chemoattractant interleukin-8, the granulocyte colony-stimulating factor (G-CSF) and the number of neutrophils are higher in an IPF lung [22], but a greater presence of neutrophils is also related to fibrosis in HP [23]. In IPF, even the number of circulating neutrophils correlated positively with the extension and the progression of fibrosis [24]. In contrast, the role of lymphocytes in ILD is contradictory, with either favorable or harmful effects depending on the proportion of Th1, Th2, Th9, Th17, Th22, or regulatory T cells [25]. Nonetheless, increased lymphocyte counts in bronchoalveolar lavage (BAL) have been associated with a significantly lower probability of disease progression in patients with non-extensive fibrosis or with non-UIP patterns [26], as well as with better outcomes in patients with acute respiratory failure [27] or acute exacerbation [28].

Corticosteroids are the most common therapy in non-IPF ILDs, while other immunomodulators (mycophenolate mofetil, cyclophosphamide, azathioprine, or tocilizumab) and antifibrotics (nintedanib and pirfenidone) are mostly reserved for CTD-ILD and IPF, respectively. Nonetheless, antifibrotics are being explored in non-IPF fibrosing ILDs (F-ILDs) [6,29,30].

The present study shows the results of BAL flow cytometry analysis at diagnosis in a large series of ILD patients, indicating that lymphocytes and neutrophils play opposing roles in the development of fibrosis, particularly in IPF, but also in the survival of patients with F-ILDs. The predictive value of these results at diagnosis could lead to better personalized immunomodulating and antifibrotic treatments.

## 2. Materials and Methods

### 2.1. Specimens

This retrospective and observational study from real-life medicine included clinical, radiological, and anatomopathological data from patients referred from public hospitals in the Murcia Region, Spain. A total of 1500 consecutive BAL samples were analyzed by flow cytometry between 2000 and 2018. Samples from patients with pulmonary infections at the moment of BAL procedure (*n* = 229), lung or other cancers (*n* = 211), asthma (*n* = 86), chronic obstructive pulmonary disease (*n* = 82), or tuberculosis (*n* = 30) were excluded. Finally, the study included 706 BAL samples from patients with ILDs. As a control BAL group, 112 BAL samples were included from patients with suspected lung disease (mainly due to the presence of micronodules on radiological images) that, after years of follow-up, did not show clinicopathological evidence of lung disease in the electronic medical record. In addition, to compare the outcome of ILD patients, 243 patients with monoclonal gammopathy of undetermined significance (MGUS), without pulmonary disease and free of adverse prognostic factors [31], were included as a survival control of the general population.

The BAL sampling procedure was performed following the official American Thoracic Society clinical practice guidelines [32].

The end of evolutionary data collection was on 1 April 2023. Anamnesis, clinical examination, radiology (radiography and high-resolution computed tomography, HRCT), BAL cytomorphology and microbiology, and anatomopathological and functional pulmonary studies were performed according to clinical practice in each hospital. Pulmonary fibrosis was computed with the presence of reticular changes, traction bronchiectasis, and honeycombing in the radiological study. IPF and other F-ILDs were studied separately. Diagnostic criteria of the ILD subtype were based on the ATS/ERS classification [33]. Immunomodulatory treatment was administered according to standard practice [34]. Antifibrotics were available from 2014 onwards with limited access according to local restrictions. Based on information from the electronic medical record, patients were grouped into 3 treatment groups: (1) patients who did not require systemic immunomodulatory treatment; (2) patients who only received corticosteroids; and (3) patients who required other immunomodulatory treatments (rituximab, azathioprine, mycophenolate mofetil, cyclophosphamide, or tacrolimus) after corticosteroids.

### 2.2. Immunophenotype Studies

**Figure 1:**
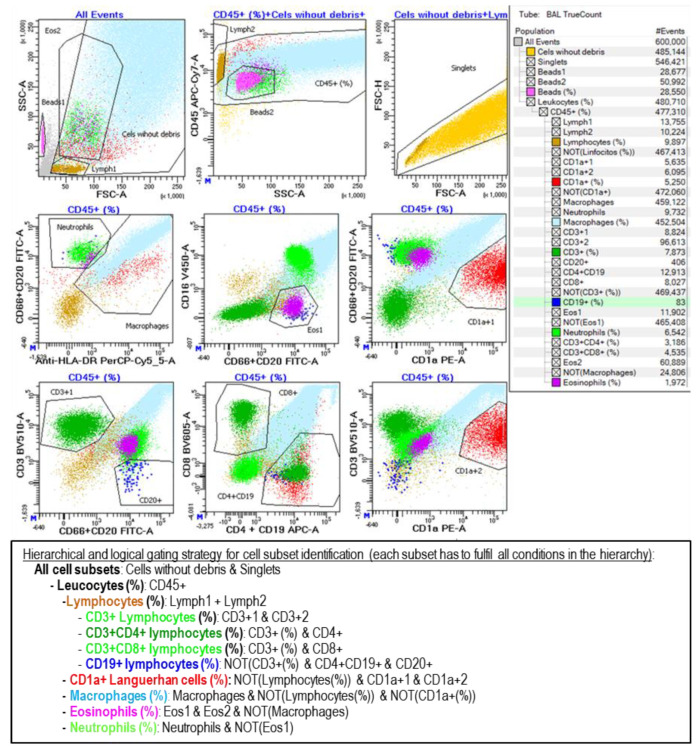
Flow cytometry analysis of the leukocyte subsets contained in BAL samples. Cell subsets were selected and colored as follows: lymphocytes in light brown (CD45++ SSClow), macrophages in cyan (DR+ SSHigh), neutrophils in light green (CD66+ CD16+ DR−), eosinophils in magenta (CD16− CD66dim), Langerhans cells in red (CD1a+), T lymphocytes in dark green (CD3+), and B lymphocytes in dark blue (CD19+ CD20+). Additionally, the CD3+CD4+ and CD3+CD8+ T subpopulations were detected. Likewise, the beads contained in the TrueCount tube were identified with a pink color, which was used to calculate the absolute cell count per microliter of the sample. Analysis gates were combined hierarchically and logically as shown on the right side and described at the bottom of the figure.

BAL samples were centrifuged, the cell pellet was washed with FACSFlow (Becton Dickinson; BD; San Jose, CA, USA) and resuspended in 0.5 mL of FACSFlow, and 50 µL of the sample was stained in a TrueCount tube (BD, San Jose, CA, USA) with the following monoclonal antibodies: CD1a-PE (HI149), CD3-BV510 (SK7), CD4-APC (SK3), CD8-PE-Cy7 (SK1), CD16-V450 (3G8), CD19-APC (SJ25C1), CD20-FITC (L27), CD45-APCH7 (2D1), and HLA-DR-PerCp (L243) from BD and CD66abce-FITC (Kat4c) from Dako (Santa Clara, CA, USA). A minimum of 0.5 million events were acquired in an 8-color FACSCanto-II flow cytometer (BD, San Jose, CA, USA) adjusted on a daily basis as previously described [31]. The analysis in Diva-Software 9.0 (BD, San Jose, CA, USA), following the gating strategy described in Figure 1, included both alive and dead cells (identified by the loss of FSC and SSC), as long as they maintained the expression of leukocyte markers.

### 2.3. Statistical Analysis

Data were collected in Excel 2010 (Microsoft Corporation, Redmond, WA, USA) and analyzed in SPSS 21.0 (Armonk, NY, USA). Kaplan–Meier and Log-Rank tests were used for survival estimation. Overall survival (OS) was defined as the time from the first BAL analysis to death, with living patients censored on the date of the last follow-up. The 75th percentile of the OS (Q3–OS) was used to compare groups. The incidence of death in each group was estimated as the number of deaths divided by the sum of the follow-up times of the patients in the group. Comparisons between quantitative variables were performed by an ANOVA. *p* < 0.05 was considered statistically significant.

## 3. Results

### 3.1. Clinical, Biological, and Therapeutic Characteristics of Study Groups

Table 1 presents the biological and clinical characteristics of the patient and control groups as well as the incidence of fibrosis (fibrosis, UIP, reticular or honeycomb patterns in the HRCT) and the main immunomodulating treatment in each pulmonary pathology. As expected, pulmonary fibrosis was present in all patients with IPF, but it was also observed in a variable proportion of other ILDs, with the highest incidence detected in NSIP (33.8%), HP (28.8%), and U-ILD (25.0%). Some pathologies were predominantly present among men, such as pneumoconiosis (96.3%), IPF (75.9%), eosinophilic ILD (76.5%), PLCH (66.7%), and LIP (64.9%). Comparable age was observed among groups, although patients with PLCH were younger (36.7 years) compared to the rest of patients (53.8 years).

### 3.2. BAL Leukocyte Profiles with a Predominance of Neutrophils Increase with Age

**Figure 2:**
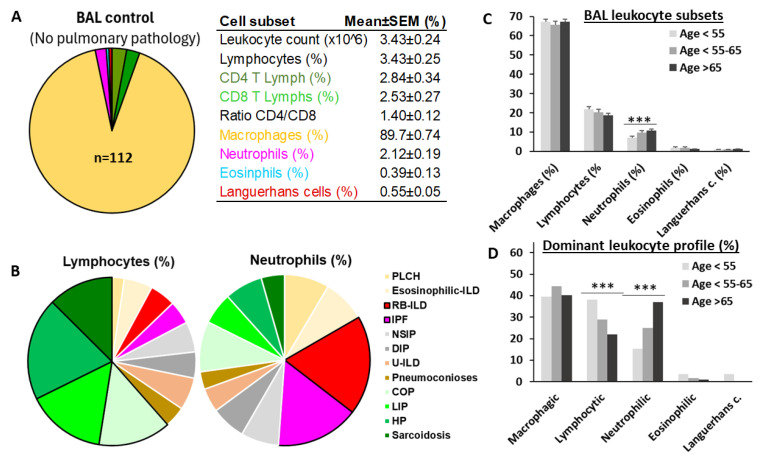
Lymphocytic ILDs decrease and neutrophilic ILDs increase with aging. (**A**) Mean values of leukocyte subset in bronchoalveolar lavage (BAL) of individuals without pulmonary pathology; (**B**) mean BAL lymphocyte and neutrophil values in patients with different types of interstitial lung disease (ILD); (**C**) mean values of macrophages, lymphocytes, neutrophils, eosinophils, and CD1a+ Langerhans cells in the BAL of total ILD patients according to age, at <55, 55–65, and >65 years; and (**D**) proportion of patients with lymphocytic (lymphocytes > 20%), neutrophilic (neutrophils > 10%), eosinophilic (eosinophils > 3%), and Langerhans cells (>1.5%) profiles, for patients aged <55, 55–65, and >65. ***, indicate *p* < 0.001 in the ANOVA test.

Figure 2A shows the leukocyte content of BAL in individuals without lung disease (Figure 2A). A predominance of macrophages (89.7 + 0.74%) and counts below 3% of the other myeloid and lymphoid subsets were observed. However, in patients with ILD, mean values of total lymphocytes were about 20% and neutrophils about 10%. Sarcoidosis, hypersensitivity pneumonitis (HP), cryptogenic organizing pneumonia (COP), and lymphocytic interstitial pneumonia (LIP) showed a lymphocytic profile (mean lymphocytes > 20.5 ± 1.3%). IPF and respiratory bronchiolitis–ILD (RB-ILD) showed neutrophilic profiles (mean neutrophils > 9.04 ± 0.7%) (Figure 2B). Although lymphocyte counts remained stable, neutrophil counts increased gradually from 7.20 ± 0.68% to 9.75 ± 1.06% and 10.73 ± 0.78% (*p* < 0.001) with the age of patients from <55 to 55–65 and >65 years, respectively (Figure 2C). As a consequence, a significant reduction in the proportion of lymphocytic ILDs (38.2%, 28.9%, and 21.9%, *p* < 0.001) and a significant increment in neutrophilic ILDs (15.4%, 25.0%, and 37.0%, *p* < 0.001) were observed as patient age increased from <55 to 55–65 and >65 years, respectively (Figure 2D).

### 3.3. Lymphocytes Counteract the Deleterious Effect of Lung-Infiltrating Neutrophils

**Figure 3:**
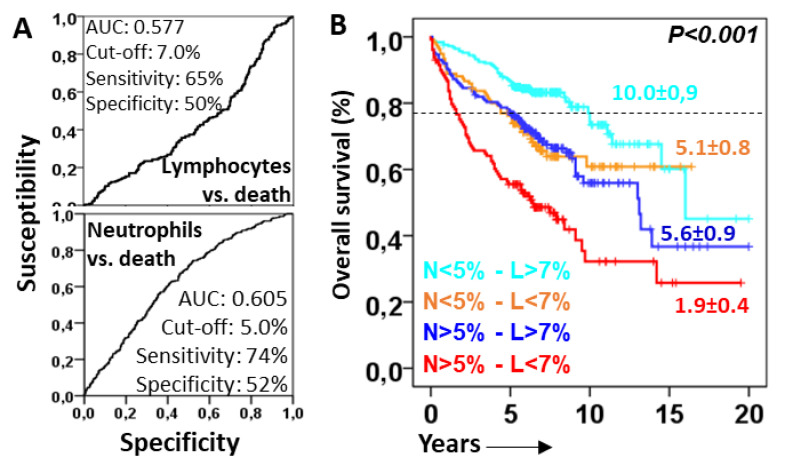
Lymphocytes protect from the deleterious effect of lung-infiltrating neutrophils. (**A**) Receiver operating characteristic curve (ROC) of lymphocytes and neutrophils related to overall survival (OS). Area under the curve (AUC) cut-offs with the highest sensitivity and specificity are shown; (**B**) Kaplan–Meier and Log-Rank tests for the OS of ILD patients according to the combinations of lymphocytes (above or below 7%) and neutrophils (above or below 5%). The 75th percentile OS is shown for each case.

ROC analysis revealed cut-offs of 7.0% and 5.0% for lymphocytes and neutrophils, respectively, associated with favorable and unfavorable prognostic values for the OS (see Figure 3A for details). The combination of neutrophil and lymphocyte cut-offs showed four groups with different survival rates (Figure 3B): patients with neutrophils < 5% and lymphocytes > 7% showed the longest Q3–OS (10.0 ± 0.9 years, *p* < 0.001); patients with neutrophils > 5% and lymphocytes < 7% showed the shortest Q3–OS (1.9 ± 0.4 years); and patients with neutrophils < 5% and lymphocytes < 7% (5.1 ± 0.8 years) or neutrophils > 5% and lymphocytes > 7% (5.6 ± 0.9 years) showed intermediate Q3–OS. Therefore, lymphocytes seemed to counteract the deleterious effect of lung-infiltrating neutrophils.

### 3.4. Lymphocytes Counteract the Harmful Effect of Neutrophils, Delaying the Onset of Lung Fibrosis and Death of ILD Patients

**Figure 4:**
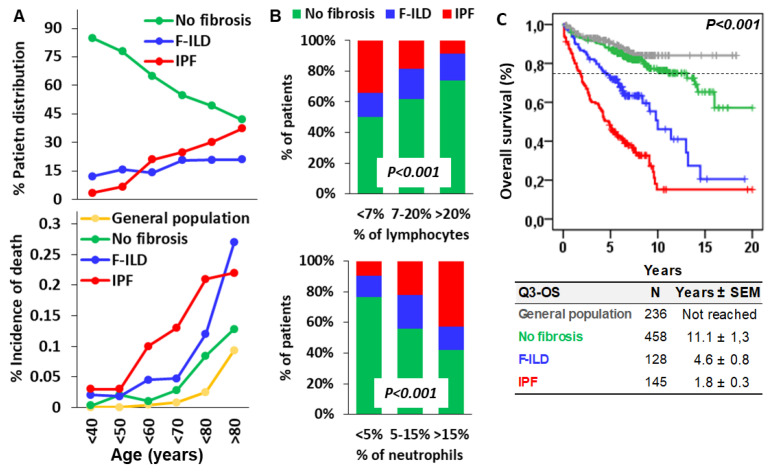
Incidence of fibrotic ILD (F-ILD), idiopathic pulmonary fibrosis (IPF), and death according to the age and the counts of lymphocytes and neutrophils in the BAL of patients. (**A**) Incidence of F-ILD, IPF, and death (estimated as the percentage of the sum of deaths divided by the sum of follow-up times of patients) according to the age of ILD patients; (**B**) incidence of F-ILD and IPF in patients according to the counts of lymphocytes (<7%, 7–20%, or >20%) and neutrophils (<5%, 5–15%, or >15%) in the BAL at diagnosis. *p* < 0.001 in the Chi-square test; and (**C**) Kaplan–Meier and Log-Rank tests for overall survival (OS) of the general population, ILD patients without fibrosis, and F-ILD and IPF patients. Pulmonary fibrosis was computed with the presence of reticular changes, traction bronchiectasis, and honeycombing in the radiological study. The 75th percentile OS (Q3–OS) is shown for each case.

As expected, the proportion of ILD patients free of pulmonary fibrosis progressively decreased from 84.8% in patients aged under 40 to 41.9% in patients aged over 90 years (Figure 4A). This was mainly due to the increasing incidence of IPF from 3.3% in patients aged under 40 to 37.2% in patients aged over 90, with the most pronounced increase between 50 and 60 years of age, from 6.7% to 21%. In contrast, the incidence of F-ILD slightly increased from 12.0% in patients under 40 to 20.9% in patients over 90. As a consequence, the incidence of death in patients with IPF was much higher at earlier ages (0.1, 0.13, and 0.21 for patients in their 60s, 70s, and 80s, respectively) than in patients with F-ILD (0.045, 0.047, and 0.12) and in patients free of fibrosis (0.01, 0.028, and 0.084) or the general population (0.004, 0.008, and 0.025). Patients with F-ILD showed a similar incidence of death to that of patients without pulmonary fibrosis until they reached an age older than 80, when they showed a higher incidence of death (0.27 vs. 0.128). Patients with ILDs free of fibrosis showed a similar incidence of death to that of the general population until their 80s (0.084 vs. 0.025) and older (0.128 vs. 0.093), when they showed a slightly higher incidence of death.

The incidence of IPF was inversely proportional to the BAL lymphocyte counts (34.1%, 18.6%, and 8.8% for lymphocytes <7%, 7–20%, and >20%, respectively), but directly proportional to BAL neutrophil counts (9.6%, 22.2%, and 42.6% for neutrophils <5%, 5–15%, and >15%, respectively). In contrast, the incidence of F-ILD was not clearly related to BAL lymphocyte or neutrophil counts (Figure 4B).

Kaplan–Meier curves showed that ILD patients without pulmonary fibrosis presented life expectancy comparable to that of the general population (Q3–OS of 11.1 ± 1.3 years vs. not reached), whilst patients with F-ILD (4.6 ± 0.8 years) and IPF (1.8 ± 0.3 years; *p* < 0.001) showed shorter survivals (Figure 4C).

Therefore, although BAL neutrophils or lymphocytes at diagnosis are related to the diagnosis of IPF but not of F-ILD, both fibrotic processes were associated with shorter lifespans of ILD patients.

**Figure 5:**
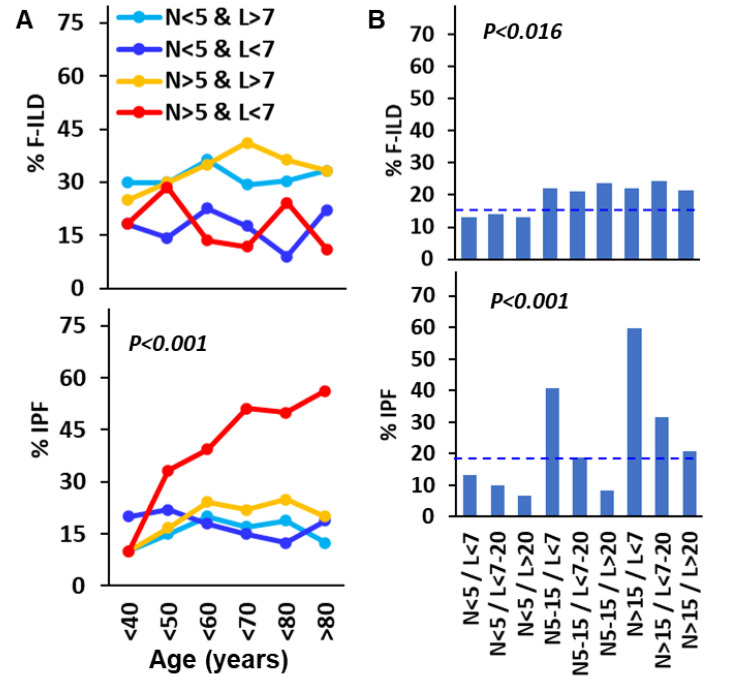
Lymphocytes counteract neutrophil-promoted idiopathic pulmonary fibrosis (IPF) during aging. (**A**) Incidence of F-ILD and IPF according to the age of ILD patients and the combined counts of neutrophils (>5%) and lymphocytes (>7%) in the BAL; (**B**) incidence of F-ILD and IPF in ILD patients according to the combined counts of neutrophils (<5%, 5–15%, or >15%) and lymphocytes (<7%, 7–20%, or >20%) in the BAL of ILD patients at diagnosis. Pulmonary fibrosis was computed with the presence of reticular changes, traction bronchiectasis, and honeycombing in the radiological study *p* estimated using the Chi-square test. Dashed lines indicate the mean incidence of fibrosis in total patients.

To further investigate the role of lymphocytes and neutrophils in the incidence of F-ILD and IPF, the combined effect of increasing amounts of neutrophils (<5%, 5–15%, and >15%) and lymphocytes (<7%, 7–20%, and >20%) were studied. Although the combined amounts of neutrophils and lymphocytes were not clearly associated with the development of fibrosis in non-IPF LDs during aging, the amounts of neutrophils above 5% and of lymphocytes below 7% were associated with increasingly higher incidences of IPF in ILD patients in their 50s and older, with incidences of IPF greater than 50% in patients in their 70s and older (*p* < 0.001). However, in patients with neutrophil counts above 5% but lymphocytes above 7%, the incidence of IPF remained between 15% and 20% in patients in their 40s to their 80s. A similar situation occurred in all patients with neutrophil counts below 5%, regardless of lymphocyte counts (Figure 5A).

When the role of BAL neutrophil and lymphocyte counts was assessed regardless of patient age, neutrophils >5% were associated with slightly higher rates of F-ILD (≈22%, *p* = 0.016) compared with neutrophils <5% (≈12%), regardless of lymphocyte counts. However, the incidence of IPF increased in parallel with neutrophil counts but was strongly counterbalanced by lymphocytes. Thus, IPF rates for neutrophil counts <5%, 5–15%, and >15% were 13.2%, 40.6%, and 59.7%, respectively, if lymphocyte counts were below 7%; but 10%, 18.8%, and 31.6% if lymphocyte counts were between 7% and 20%; or 6.5%, 8.4%, and 20.7% if lymphocyte counts were over 20% (*p* < 0.001). This demonstrates a dose–response effect both of neutrophils in inducing IPF and of lymphocytes in protecting against the deleterious effect of neutrophils (Figure 5B).

### 3.5. Neutrophil and Lymphocyte Counts Were Associated with Patient Overall Survival in Non-IPF ILD, but Not in IPF

**Figure 6:**
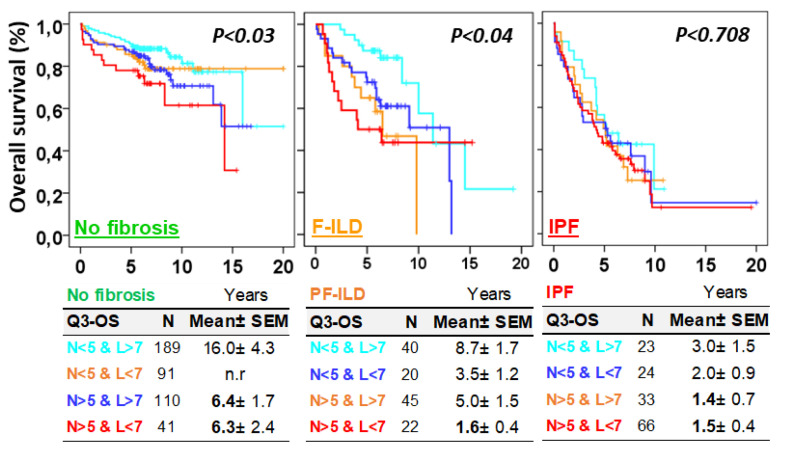
Neutrophil and lymphocyte counts were associated with the overall survival (OS) of ILD patients without fibrosis and with pulmonary fibrosis associated with ILDs (F-ILDs), but not with the OS of patients with idiopathic pulmonary fibrosis (IPF). Kaplan–Meier and Log-Rank tests for the OS of ILD patients according to the combinations of lymphocytes (above or below 7%) and neutrophils (above or below 5%) and the type of fibrotic process (F-ILD or IPF). Pulmonary fibrosis was computed with the presence of reticular changes, traction bronchiectasis, and honeycombing in the radiological study.

Next, we evaluated the influence of the combined neutrophil and lymphocyte counts on the patient’s overall survival. In patients without fibrosis or with F-ILD, neutrophil and lymphocyte counts were negatively and positively associated with patient survival, respectively. Thus, the Q3–OS for patients with neutrophils < 5% and lymphocytes > 7%, neutrophils < 5% and lymphocytes < 7%, neutrophils > 5% and lymphocytes > 7%, and neutrophils > 5% and lymphocytes < 7% for patients without fibrosis were 16.0 ± 4.3, not reached, 6.4 ± 1.7, and 6.4 ± 2.4 years (*p* = 0.03), respectively; and for patients with F-ILD, the values were 8.7 ± 1.7, 3.5 ± 1.2, 5.0 ± 1.5, and 1.6 ± 0.4 years (*p* = 0.04), respectively. However, in IPF patients, once the disease was diagnosed, survival was much shorter regardless of the neutrophil and lymphocyte counts (*p* = 0.708, Figure 6).

### 3.6. The Type of Immunomodulatory Treatment Differentially Influenced Overall Survival in Patients With or Without Fibrosis

**Figure 7:**
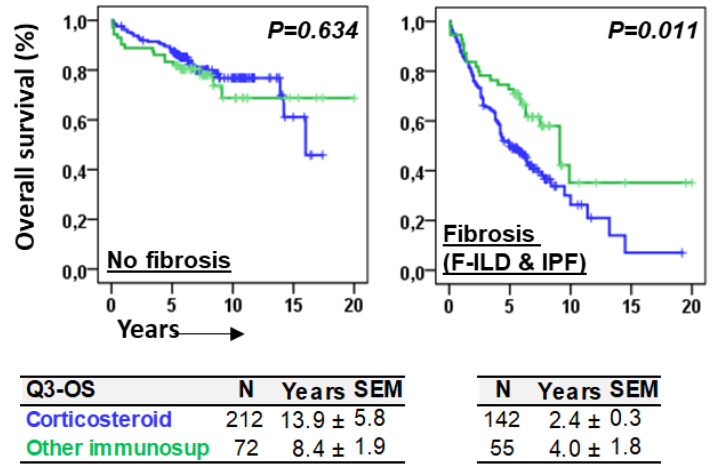
Immunomodulating therapies were differentially associated with overall survival in patients with or without fibrosis. Kaplan–Meier and Log-Rank tests for the overall survival (OS) of ILD patients according to the type of immunomodulatory treatment, corticosteroids vs. other immunosuppressants (rituximab, azathioprine, mycophenolate mofetil, cyclophosphamide, or tacrolimus), and the presence of fibrosis. The 75th percentile of the OS (Q3–OS) is shown for each case.

The type of immunomodulating therapies, consisting mainly of corticosteroids or other types of immunosuppressants (rituximab, azathioprine, mycophenolate mofetil, cyclophosphamide, or tacrolimus), was not associated with the differential OS of patients without fibrosis (*p* = 0.634). However, patients with fibrosis (F-ILD and IPF) treated with corticosteroids showed a shorter Q3–OS (2.4 ± 0.3 vs. 4.0 ± 1.9 years, *p* = 0.011) than patients treated with other immunosuppressants (Figure 7).

## 4. Discussion

Although the course of the disease for an IPF patient cannot currently be predicted at diagnosis, data from clinical trials and observational studies indicate that antifibrotic therapies can improve life expectancy [14]. However, many individuals with IPF remain untreated, either because the physician perceives that the disease is stable or because they are concerned about its potential side effects [35]. Early treatment is critical to preserving patients’ lung function, reducing the risk of acute exacerbations, and improving outcomes of this disease that induce irreversible and fatal damage. Data from this manuscript and others [36] make it clear that once IPF is diagnosed, the survival of these patients in relation to the general population or other ILDs is drastically shortened. Therefore, the availability of adequate prognostic markers could help clinicians to prescribe antifibrotic treatments. In this sense, our data can support early antifibrotic treatment since young patients with a greater probability of developing fibrosis or having more rapidly progressive fibrosis are identified at diagnosis, due to the mere fact that they have a high number of neutrophils infiltrating the lung without the control that lymphocytes exert on these harmful cells. However, the fact that current clinical guidelines indicate that patients with a clear UIP pattern do not require BAL analysis poses a challenge [32], as it could help with prognosis, therapy guidance, and the discrimination of overlapping UIP patterns in IPF and other ILDs [30]. In fact, in our series, up to 14% of IPF patients with lymphocytes >20% were detected in BAL. In these patients, the analysis of the cellular content of BAL could have helped to diagnose this pathology more correctly [37].

It has been described that alveolar macrophages play an important antifibrotic role and contribute to alveolar homeostasis [36], so it could be thought that any reduction in their numbers could be associated with fibrosis. Our results show that as long as this reduction is made by neutrophils, a profibrotic state is established at an early age; however, when this reduction is due to lymphocytes, the fibrotic processes are attenuated. More importantly, when both neutrophils and lymphocytes are elevated, the presence of lymphocytes was associated with a reduction in the fibrosis rates in a dose–response manner. Although there is no clear evidence that lymphocytes may protect patients from fibrosis, beyond the role described for type-II innate lymphocytes (ILCs2) [38], our data seem to demonstrate that the infiltration of the lung by lymphocytes might be associated with less fibrosis. This fact could be connected with the longer OS observed in patients with fibrosis treated with non-corticosteroid immunomodulators [14], also observed in our series.

IPF and F-ILDs have overlapping genetics, pathophysiological mechanisms, and clinical behavior [30,39]. However, the data described in this study show that the results of lung-infiltrating lymphocytes or neutrophils are different in these pathologies. Although, in both clinical entities, high counts of lymphocytes and neutrophils had either favorable or unfavorable predictive value, respectively; in IPF, these cells were critical for an earlier development of the disease but were not so clearly associated with the patient’s overall survival once the disease was diagnosed. In contrast, the development of F-ILD did not seem to be clearly related to lymphocyte and neutrophil counts; nonetheless, high neutrophil counts were associated with shorter overall survival and a greater amount of lymphocytes appeared to decrease the negative contribution of neutrophils. Although the beneficial effect of lymphocytes could be due to a better response to immunomodulating treatments in lymphocytic lung pathologies [37,40], the fact is that patients with both high numbers of neutrophils and high numbers of lymphocytes were associated with a lower rate of IPF and longer survival in F-ILDs. Therefore, the identification of factors secreted by lymphocytes or their interactions in the lung microenvironment [36] could contribute to the development of new therapies to mitigate fibrogenesis and improve patient outcomes.

Unfortunately, in this retrospective study it has not been possible to establish the progressive condition in all F-ILD cases, so we cannot know whether neutrophils and lymphocytes may be involved in the development of progressive F-ILD. However, the shorter survival in both fibrotic and non-fibrotic ILDs associated with high counts of neutrophils, improved with higher lymphocyte counts, suggests that these cells, like in IPF, may be involved in the development of progressive F-ILD. This should be evaluated in new and future studies. In addition, our study was also unable to assess the impact of modern antifibrotics, due to the limited availability of this treatment during the study period. However, conducting clinical trials evaluating antifibrotics in progressive F-ILDs [29,30] would benefit from the analysis of BAL cell content as their results could be different depending on the inflammatory microenvironment of the lung.

In conclusion, the results of our study suggest that accurate counting of BAL lymphocytes and neutrophils by flow cytometry in ILD patients at diagnosis could help guide immunomodulatory and antifibrotic therapies. However, further studies will be necessary to confirm our results and identify the molecular mechanisms involved.

## Figures and Tables

**Table 1:** Biological, clinical, and therapeutic characteristics of patient and control groups.

	Patients (*n*)	Sex (% Man)	Age (Mean ± SD)	Fibrosis ^1^ *n* (%)	Incidence of Death ^2^	Treatments (%) ^3^			
						NT	IC	SC	Other
Control groups	343	53.2%	61.2 ± 13.5		0.023				
General population ^4^	243	53.1%	67.9 ± 12.3		0.021				
BAL control ^5^	112	52.70%	53.5 ± 16.6		0.027				
Idiopathic pulmonary fibrosis (IPF)	145	75.90%	66.4 ± 10.9	145 (100%)	0.147	36	31	51	27
Other interstitial lung diseases (ILDs)	561	54.60%	57.3 ± 17.1	125 (22.3%)	0.035	189	93	177	99
Sarcoidosis	82	47.60%	55.6 ± 14.8	16 (19.5%)	0.016	28	11	31	12
Hypersensitivity pneumonitis (HP)	48	54.20%	49.7 ± 17.6	15 (28.8%)	0.032	6	13	22	6
Organized cryptogenic pneumonia (COP)	44	43.20%	62.3 ± 17.4	8 (18.6%)	0.048	8	4	23	9
Lymphocytic interstitial pneumonia (LIP)	37	64.90%	53.2 ± 15.9	3 (7.9%)	0.025	18	4	9	6
Respiratory bronchiolitis ILD (RB-ILD)	25	40.0%	53.3 ± 22.8	2 (8%)	0.029	9	5	9	2
Desquamative interstitial pneumonia (DIP)	35	51.40%	54.4 ± 20.8	2 (5.6%)	0.035	16	5	8	6
Nonspecific interstitial pneumonia (NSIP) ^6^	156	49.40%	59.3 ± 15.5	52 (33.8%)	0.044	48	18	43	47
Pneumoconiosis	27	96.3%	56.5 ± 16.0	5 (19.2%)	0.052	11	8	6	1
Pulmonary Langerhans c. histiocytosis (PLCH)	9	66.7%	36.7 ± 15.8	1 (11.1%)	0.033	6	1	1	1
Eosinophilic ILD	17	76.50%	51.2 ± 22.7	1 (5.9%)	0.030	0	3	13	1
Unclassifiable ILD (U-ILD)	80	60.0%	61.3 ± 12.4	20 (25%)	0.038	39	21	12	8

^1^ Pulmonary fibrosis was computed with the presence of reticular changes, traction bronchiectasis, and honeycombing in the radiological study. ^2^ Number of deaths divided by the sum of follow-up years of patients in each group. ^3^ NT: no treatment; IC: inhaled corticoid; SC: systemic corticoid. Other immunosuppressants: rituximab, azathioprine, mycophenolate mofetil, cyclophosphamide, or tacrolimus. ^4^ Patients with monoclonal gammopathy with a good prognosis (without cytogenetic alterations or tumor circulating plasma cells in the peripheral blood). ^5^ BAL was performed for etiological affiliation but with no evident ILD pathology during follow-up. ^6^ Patients with connective tissue disease–ILD were included mostly in this group.

## Data Availability

The data that support the findings of this study are available from the corresponding author upon reasonable request.
